# Evaluation of an Amino Acid Mix on the Secretion of Gastrointestinal Peptides, Glucometabolic Homeostasis, and Appetite in Obese Adolescents Administered with a Fixed-Dose or ad Libitum Meal

**DOI:** 10.3390/jcm9093054

**Published:** 2020-09-22

**Authors:** Antonello E. Rigamonti, Sofia Tamini, Sabrina Cicolini, Alessandra De Col, Diana Caroli, Stefania Mai, Eugenia Rondinelli, Antonella Saezza, Silvano G. Cella, Alessandro Sartorio

**Affiliations:** 1 Department of Clinical Sciences and Community Health, University of Milan, 20129 Milan, Italy; silvano.cella@unimi.it; 2 Experimental Laboratory for Auxo-endocrinological Research, Istituto Auxologico Italiano, IRCCS, 28824 Verbania, Italy; s.tamini@auxologico.it (S.T.); s.cicolini@auxologico.it (S.C.); a.decol@auxologico.it (A.D.C.); d.caroli@auxologico.it (D.C.); sartorio@auxologico.it (A.S.); 3 Experimental Laboratory of Metabolic Research, Istituto Auxologico Italiano, IRCCS, 28824 Verbania, Italy; s.mai@auxologico.it; 4 Laboratory of Clinical Analyses, Istituto Auxologico Italiano, IRCCS, 28824 Verbania, Italy; e.rondinelli@auxologico.it; 5 Division of Auxology, Istituto Auxologico Italiano, IRCCS, 28824 Verbania, Italy; a.saezza@auxologico.it

**Keywords:** amino acids, appetite, gastrointestinal peptides, obesity

## Abstract

Proteins have been demonstrated to reduce food intake in animals and humans via peripheral and central mechanisms. Supplementation of a dietetic regimen with single or mixed amino acids might represent an approach to improve the effectiveness of any body weight reduction program in obese subjects. The aim of the present study was to evaluate the effects of an amino acid mix (L-arginine + L-leucine + L-glutamine + L-tryptophan) on the secretion of some gastrointestinal peptides (i.e., ghrelin and glucagon-like peptide type 1, GLP-1), glucometabolic homeostasis (i.e., glucose, insulin, and glucagon), and appetite (hunger/satiety scored by visual analogue scale, VAS) in obese adolescents (*n* = 14; 10 females and 4 males; age: 16.6 ± 1.0 years; body mass index (BMI): 36.4 ± 4.6 kg/m²; fat-free mass (FFM): 54.9 ± 4.7%; fat mass (FM): 45.1 ± 4.4%) administered with a fixed-dose (lunch) or ad libitum (dinner) meal. Isocaloric maltodextrins were used as control treatment. During the lunch test, a significant increase in circulating levels of GLP-1, but not of ghrelin, was observed in the amino acid-treated group, which was congruent with significant changes in appetite, i.e., increase in satiety and decrease in hunger. A significant hyperglycemia was found in the maltodextrin-treated group during the prelunch period, without any significant changes in insulin and glucagon between the two groups. During the dinner test, there were no significant differences in appetite (hunger/satiety) and intake of calories. In conclusion, L-arginine, L-leucine, L-glutamine, and L-tryptophan, when administered to obese adolescents with a fixed-dose meal, are capable of evoking an anorexigenic response, which is, at least in part, mediated by an increase in GLP-1 released in circulation by L cells, which are capable of chemosensing specific amino acids present in the intestinal lumen. Further additional studies are requested to understand whether higher doses are necessary to inhibit ad libitum feeding.

## 1. Introduction

The satiating effect of proteins is recognized to be higher than that of other macronutrients [1,2,3,4,5]. Protein-based diets are reported to reduce food intake, promote weight loss, and improve body composition in animals and humans [6,7,8,9,10,11].

There is evidence that proteins influence the release of some gastrointestinal peptides implicated in the control of energy balance, including appetite [12,13]. In particular, some studies have shown that food intake increases circulating levels of some anorexigenic gastrointestinal peptides, such as glucagon-like peptide type 1 (GLP-1) and peptide YY (PYY) [14,15,16,17]. Notably, if the meal is rich in proteins, there is a higher increase in circulating levels of GLP-1 and PYY in normal-weight subjects than if the meal is rich in carbohydrates or fats [18]. Furthermore, knockout murine models, such as PYY^−/−^ mice, have been shown to be resistant to the hypophagic effect of a protein-based diet [15].

Our gut is endowed with a sophisticated “nutrient-sensing” system, which consists of a heterogeneous family of chemoreceptors that is expressed in specialized enteroendocrine cells and capable of recognizing specific nutrients present in the lumen after ingestion/digestion of a meal [19,20]. Though representing less than 1% of the intestinal epithelial lining, enteroendocrine cells play a pivotal role in energy balance by chemosensing nutrients, secreting gastrointestinal peptides in circulation, and ultimately regulating food intake at the hypothalamic level [21,22]. For instance, L cells, enteroendocrine cells mainly localized in the large intestine, are implicated in the synthesis/release of GLP-1 and PYY, evoking an anorexigenic response after ingestion/digestion of a meal [23,24].

Amino acids deriving from the digestion of proteins present in our diet may be chemosensed not only at the peripheral level by enteroendocrine cells but also at the central level by hypothalamic neurons [12]. In this context, single or mixed amino acids have been shown to influence the secretion of some gastrointestinal peptides and, consequentially, regulate appetite [25,26]. Chemoreceptors have been identified to bind and recognize specific amino acids belonging to class C G-protein-coupled receptors, some of which are expressed in L cells [21]. These receptors, including the calcium-sensing receptor (CaSR), the heterodimeric taste receptor T1R1/T1R3 (taste 1 receptor member 1 / taste 1 receptor member 3), and the receptor GPRC6A (G protein-coupled receptor family C group 6 member A), are defined as “promiscuous” because they bind to different structurally related L-amino acid ligands, which can derive from protein digestion (by pancreatic proteases or microbiota enzymes) [19,27]. 

Supplementation of food with specific amino acids, selected for their ability to bind to these chemoreceptors and for their effect of inhibiting food intake, might represent a new approach for the prevention and/or treatment of obesity [28]. In this regard, modern nutraceutics should be applied to obesiology with the aim of supplementing a dietetic regimen with nutrients that inhibit appetite (decrease hunger or increase satiety), thereby reducing food intake and weight gain [29,30].

The anorexigenic properties of single amino acids are very different [12]. One of most studied amino acid in animals and humans is L-arginine, defined as “satiating amino acid”. In fact, it is a conditionally essential amino acid that can activate many chemoreceptors, including CaSR, GPRC6A, and T1R1/T1R3, with the latter being the most important [31]. L-arginine reduces food intake and increases circulating levels of GLP-1 and PYY in animals [32,33,34]. Recently, L-arginine has been reported to be ineffective in fasting individuals but to increase secretion of PYY and GLP-1 after administration of an ad libitum meal, without changing appetite and calorie intake [35]. L-glutamine stimulates secretion of GLP-1, so as to be defined as amino acid “GLP-1-secretagogue” [36,37,38]. L-leucine [39] and L-tryptophan [40] are endowed with similar anorexigenic properties.

Based on the previous premises, the aim of the present study was to evaluate the effects of an amino acid mix, namely, L-arginine, L-leucine, L-glutamine, and L-tryptophan, administered at “physiological” doses (i.e., similar to the amount present in a meal considered at high protein content) on the secretion of some gastrointestinal peptides, particularly (the anorexigenic) GLP-1 and (the orexigenic) ghrelin, glucometabolic homeostasis (i.e., glucose, insulin, and glucagon), and appetite (hunger/satiety) in a group of obese adolescents during a fixed-dose or ad libitum meal. The reason for recruiting young subjects derives from the pandemic spread of pediatric obesity and the urgent need for early intervention with “effective” body weight reduction programs in obese children/adolescents [41].

## 2. Material and Methods

### 2.1. Patients and Experimental Protocol

**Figure 1:**
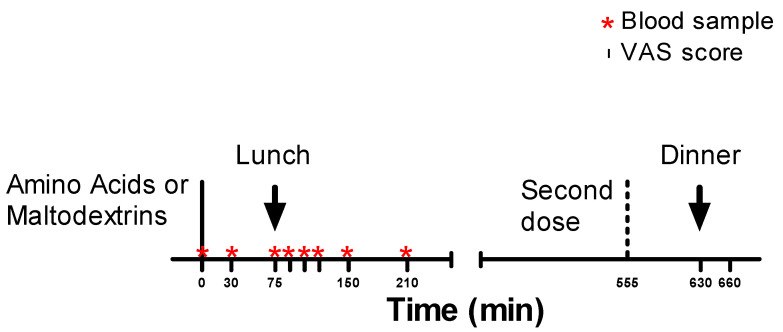
Overview of the experimental protocol. VAS: visual analogue scale.

A total of 14 obese adolescents (10 females and 4 males; age: 16.6 ± 1.0 years; body mass index (BMI): 36.4 ± 4.6 kg/m²; fat-free mass (FFM): 54.9 ± 4.7%; fat mass (FM): 45.1 ± 4.4%) were recruited among patients hospitalized for a multidisciplinary body weight reduction program at the Division of Auxology, Istituto Auxologico Italiano, IRCCS, Piancavallo (VB, Verbania, Italy). The study was completed in the first days of hospitalization in order to avoid any carry-over effect due to weight, diet, and physical activity changes. Subjects having any disease apart from morbid obesity or taking any drug were excluded. Furthermore, the weight needed to be stable (not more than 3 kg change in the previous month) to take part in the study. All females were eumenorrheic. The protocol is summarized in Figure 1.

In separate days, with a wash-out period of at least 7 days, in agreement with a randomized order and cross-over design, starting from 8.00 a.m., the participants underwent two tests consisting of oral administration, after 12 h of overnight fasting, of a drink containing an amino acid mix (3 g of L-arginine + 3 g of L-tryptophan + 6 g of L-leucine + 6 g of L-glutamine, for a total of 306 kJ) or placebo (18.7 g of maltodextrins, Enervit Maltodestrine Sport, Enervit spa, Erba, Italy, for a total of 306 kJ). The choice of the amino acid doses was based on pharmacodynamic and pharmacokinetic considerations of clinical studies previously published [35,36,37,38,39,40]. The powder of each supplement (amino acids or maltodextrins) was dissolved in 150 mL of orange juice (with 0.75 g of proteins, 13.1 g of carbohydrates, and 0.0 g of fats, for a total of 230 kJ). The drink was consumed within 9 min (50 mL every 3 min three times). Each drink had the same color (yellow) and taste (orange) in order to avoid possible visual and taste conditioning. After an hour and a half (i.e., at 75 min, T75), a prepackaged pizza margherita (350 g with 17.5% protein, 49% carbohydrate, and 33.5% fat, for a total of 3347 kJ), identical for both tests, was offered to all subjects, who were asked to completely consume the meal within 15 min. During the lunch, drinking still water was permitted (max. 250 mL).Blood samples were drawn from all participants starting from T0 (baseline, before administering the drink) until T210, for a total of 8 samples, i.e., T0 (0 min), T30 (30 min), T75 (75 min), T90 (90 min), T105 (105 min), T120 (120 min), T150 (150 min), and T210 (210 min). Using a visual analogue scale (VAS), appetite (satiety and hunger) was evaluated at the following times: T0 (0 min), T30 (30 min), T75 (75 min), T90 (90 min), T105 (105 min), T120 (120 min), T150 (150 min), and T210 (210 min). In particular, subjects were asked to rate their satiety and hunger on a 10 cm line, with labels at the extremities indicating the most negative and the most positive ratings.

At 5.15 p.m. (i.e., T555, 555 min), participants were administered a second dose of the same treatment (amino acids or maltodextrins). After 75 min (i.e., at 6.30 p.m., T630, 630 min), a semisolid nonpalatable dinner (mashed potatoes and vegetables consisting of 10% protein, 60% carbohydrate, and 30% fat) was offered. The meal was consumed ad libitum up to a maximum corresponding to 80% of the individual daily caloric intake. The meal time was 20 min. Before starting the dinner and half an hour after (at T660, 660 min), hunger and satiety were measured by VAS. At the end of dinner, the residual amount of food was recorded, and the corresponding caloric content was calculated. If the subject had eaten food accounting for more than 100% of the individual daily energy intake recommended by the dietician for the in-hospital body weight reduction program, a compensatory caloric restriction was imposed during meals of the following days.

The study protocol was approved by the Ethical Committee of Istituto Auxologico Italiano (research project code: 01C924; acronym: MISCEAMINOB). All subjects and their parents gave their written consent after being fully informed about every aspect of the study protocol.

### 2.2. Evaluation of Body Composition

Anthropometric characteristics were evaluated during the screening period. BMI was calculated from measured height and weight. The evaluation of FFM and FM was performed throughout bioimpedentiometry (Human-IM Scan, DS-Medigroup, Milan, Italy).

### 2.3. Blood Sampling and Biochemical Measurements

Blood was collected in tubes with or without anticoagulant ethylenediaminetetraacetic acid (EDTA). Plasma or serum was separated by centrifugation and stored at −20 °C.

Total plasma ghrelin level, including both octanoylated and des-octanoylated ghrelin, was measured by a commercially available ELISA kit for ghrelin (Millipore, Saint Charles, MO, USA). The sensitivity of the method was 50 pg/mL; intra- and interassay coefficients of variation (CVs) were 1.26% and 7.81%, respectively. Plasma glucagon level was measured by a commercially available radioimmunoassay (RIA) kit for glucagon (OMNIA DIAGNOSTICA srl, Misterbianco, CT, Italy). The sensitivity of the method was 25 pg/mL, and the intra- and interassay CVs were <12.3% and <12.4%, respectively.

Total plasma GLP-1 level, including GLP-1_7-36 amide_, GLP-1_7-37_, GLP-1_9-36 amide_, GLP-1_9-37_, GLP-1_1-36 amide_, and GLP-1_1-37_, was determined by an ELISA kit (Millipore, Saint Charles, MO, USA). A DPP-IV (dipeptidyl protease IV) inhibitor (protease inhibitor cocktail, Sigma Aldrich-Merck, Darmstadt, Germany) was added to tubes (50 µL) in order to prevent breakdown of GLP-1. The sensitivity of the method was 1.5 pmol/L, and the intra- and interassay CVs were 1% and <12%, respectively. Serum insulin concentration was determined by a chemiluminescent immunometric assay using a commercial kit (Immulite 2000, DPC, Los Angeles, CA, USA). The sensitivity of the method was 2 µIU/mL, and the intra- and interassay CVs were 22%–38% and 14%–23%, respectively.

Serum glucose level was determined by the glucose oxidase enzymatic method (Roche Diagnostics, Monza, Italy).

### 2.4. Statistical Analyses

The Sigma Stat 3.5 statistical software package was used for data analyses, while GraphPad Prisma 5.0 software was used for plotting data.

In order to determine a priori the sample size, a power analysis was performed by considering a difference in mean values of circulating levels of GLP-1 at T90 after amino acids vs. maltodextrins equal to 20.0 ± 18.0 pmol/L, with an α error of 0.05 at two tails and a power of 0.80. The Shapiro–Wilk test showed that all parameters were normally distributed.

Results are reported as mean ± standard deviations (SD). The responses in glucose, insulin, ghrelin, glucagon, GLP-1, and VAS scores for hunger and satiety were evaluated as absolute values for each experimental group (amino acids vs. maltodextrins).

All parameters (ghrelin, glucagon, GLP-1, VAS scores for hunger and satiety, glucose, and insulin) were compared within each experimental group (amino acids or maltodextrins) over sampling times vs. T0 (intragroup analysis) and between the two experimental groups (amino acids vs. maltodextrins) for any sampling time (intergroup analysis) using a two-way ANOVA with repeated measures (with the two factors time and group and the interaction time × group), followed by the post hoc Tukey’s test (T0–T210, lunch test with administration of a fixed-dose meal). The same statistical method was used to compare postdinner responses in VAS scores for hunger and satiety vs. T630 (intragroup analysis) and among subjects administered with amino acids vs. maltodextrins (intergroup analysis) (T630–T660, dinner test with administration of ad libitum meal). The difference in intake of calories between the two groups (amino acids vs. maltodextrins) at dinner was evaluated using the Student’s *t*-test for paired data. Significance was set at a level of *p* < 0.05 for all data analyses.

## 3. Results

**Figure 2:**
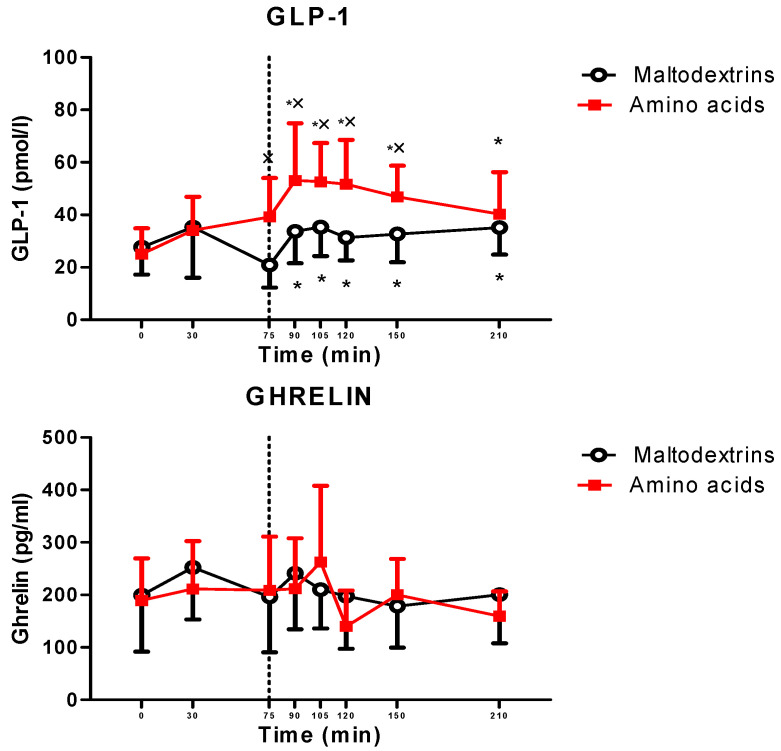
Changes in circulating levels of glucagon-like peptide type 1 (GLP-1 (top panel)) and ghrelin (bottom panel) in obese adolescents after a fixed-dose lunch (completely consumed within 15 min starting at T75), administered with amino acid mix (L-arginine, L-leucine, L-glutamine, and L-tryptophan) or placebo (maltodextrins) at T0. See the text for further details. Values are expressed as means ± standard deviations (SDs). The number of subjects was 14. * *p* < 0.05 vs. the corresponding T0 value; × *p* < 0.05 vs. the value in the maltodextrin-treated group at the corresponding time point. A two-way ANOVA with repeated measures (with the two factors time and group and the interaction time × group) followed by the post hoc Tukey’s test was used.

The lunch significantly increased GLP-1 (at T90, T105, T120, T150, and T210 vs. T0 for amino acids and maltodextrins, *p* < 0.05), while it did not modify ghrelin levels. The postprandial responses of GLP-1 were significantly different between the two experimental groups (amino acids vs. maltodextrin at T75, T90, T105, T120, and T150, *p* < 0.05), while those of ghrelin were not significantly different (Figure 2).

**Figure 3:**
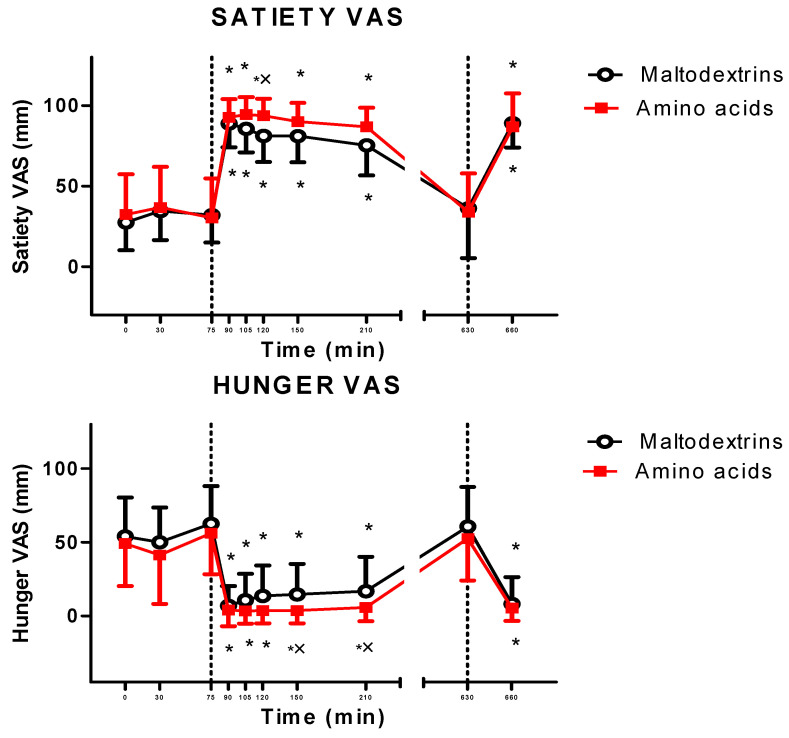
Changes in VAS ratings of satiety (top panel) and hunger (bottom panel) in obese adolescents after a fixed-dose lunch (completely consumed within 15 min starting at T75) or ad libitum dinner (within 20 min starting at T630), administered with amino acid mix (L-arginine, L-leucine, L-glutamine, and L-tryptophan) or placebo (maltodextrins) at T0 for the lunch test and at T555 for the dinner test. See the text for further details. Values are expressed as means ± SDs. The number of subjects was 14. * *p* < 0.05 vs. the corresponding T0 or T630 value; × *p* < 0.05 vs. the value in the maltodextrin-treated group at the corresponding time point. A two-way ANOVA with repeated measures (with the two factors time and group and the interaction time × group) followed by the post hoc Tukey’s test was used.

The lunch significantly increased and decreased satiety and hunger, respectively (satiety: at T90, T100, T120, T150, and T210 vs. T0 for amino acids and maltodextrins, *p* < 0.05; hunger: at T90, T105, T120, T150, and T210 vs. T0 for amino acids and maltodextrins, *p* < 0.05). Postprandial responses in hunger and satiety were significantly different among subjects treated with amino acids and maltodextrins (amino acids vs. maltodextrins at T120 for satiety and at T150 and T210 for hunger, *p* < 0.05) (Figure 3).

**Figure 4:**
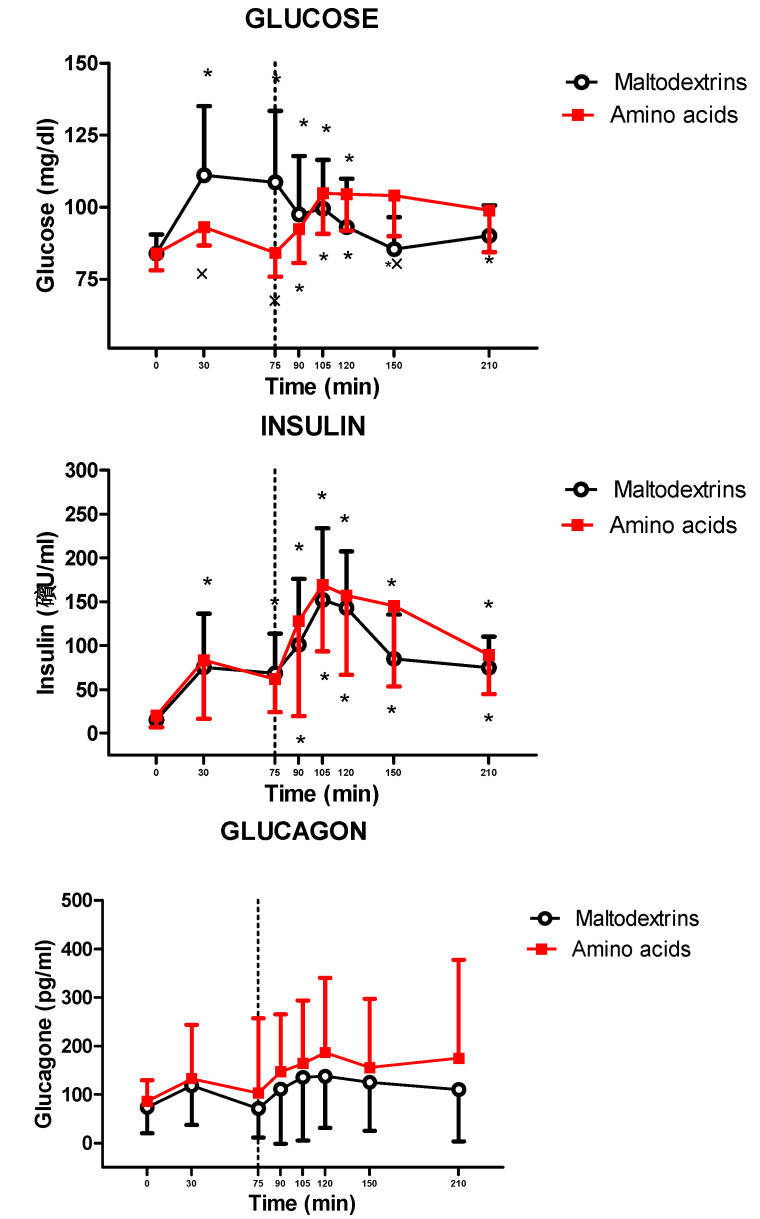
Changes of circulating levels of glucose (top panel), insulin (middle panel), and glucagon (bottom panel) in obese adolescents after a fixed-dose lunch (completely consumed within 15 min starting at T75) administered with amino acid mix (L-arginine, L-leucine, L-glutamine, and L-tryptophan) or placebo (maltodextrins) at T0. See the text for further details. Values are expressed as means ± SDs. The number of subjects was 14. * *p* < 0.05 vs. the corresponding T0 value; × *p* < 0.05 vs. the value in the maltodextrin-treated group at the corresponding time point. A two-way ANOVA with repeated measures (with the two factors time and group and the interaction time × group) followed by the post hoc Tukey’s test was used.

The lunch significantly increased glucose and insulin levels (glucose: at T90, T105, T120, T150, and T210 vs. T0 for amino acids and at T30, T75, T90, T105, and T120 vs. T0 for maltodextrins, *p* < 0.05; insulin: at T90, T105, T120, T150, and T210 vs. T0 for amino acids and at T30, T75, T90, T105, T120, T150, and T210 vs. T0 for maltodextrins, *p* < 0.05), while it did not modify glucagon levels. Postprandial responses of glucose were significantly different among subjects treated with amino acids and maltodextrins (amino acids vs. maltodextrins: at T30, T75, and T150, *p* < 0.05), while those of insulin and glucagon were not significantly different (Figure 4).

**Figure 5:**
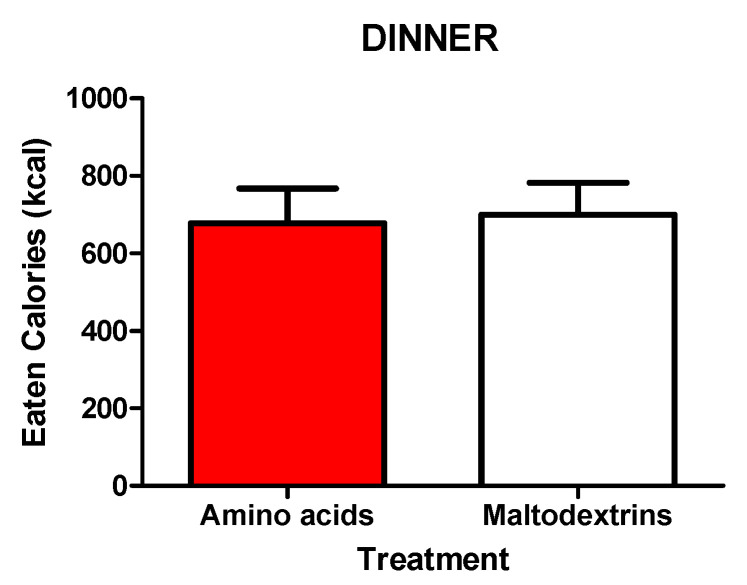
Intake of calories of food (i.e., difference between pre- and postdinner values) in obese adolescents after ad libitum dinner (consumed within 20 min starting at T555), administered with amino acid mix (L-arginine, L-leucine, L-glutamine, and L-tryptophan) or placebo (maltodextrins) at T0. See the text for further details. Values are expressed as means ± SDs. The number of subjects was 14. A Student’s *t-*test for paired data was used.

An hour and a half after the administration of the second dose of the treatment (amino acids or maltodextrins), the dinner significantly increased satiety and decreased hunger, respectively (T630 vs. T660, *p* < 0.05), with no significant differences between the two experimental groups (Figure 3). The amount of calories consumed during dinner did not differ among subjects treated with amino acids vs. maltodextrins (Figure 5).

## 4. Discussion

The present study carried out in a group of obese adolescents shows that supplementation with an amino acid mix, namely, L-arginine, L-leucine, L-glutamine, and L-tryptophan, was capable of reducing appetite, particularly determined by a decrease in hunger and an increase in satiety, when a fixed-dose meal was offered. This effect, though modest, was mediated by an increase in GLP-1 levels in the amino acid-treated group, while the role of ghrelin was negligible. The increase in GLP-1 levels by amino acids already occurred at T75, before starting the ingestion of lunch.

Our hypothesis is that the selected amino acids, i.e., L-arginine, L-leucine, L-glutamine, and L-tryptophan, directly stimulated L cells localized within the mucosa of the gastrointestinal tract [19,42] by activating some chemoreceptors, such as CaSR, T1R1/T1R3, and GPRC6A [20,21], with the ensuing secretion of GLP-1 in circulation [24]. GLP-1 being an anorexigenic peptide acting at the hypothalamic level [43], subjects administered with amino acids would have experienced reduced appetite after lunch consisting of a fixed-dose meal, an experimental paradigm that was adopted to eliminate the confounding factor of a different intake of calories (as in ad libitum feeding) on gastrointestinal peptides [44].

Generally, GLP-1 increases satiety rather than decreasing hunger [43]. Although it is neuropsychologically and neurobiologically difficult to separate decreased hunger from increased satiety, due to overlapping neuroanatomical areas and neuropharmacological regulations [45], in the present study, the increase in satiety appeared earlier (at T90) than the decrease in hunger (at T120 and T150). This might denote the involvement of other orexigenic peptides different from the unchanged ghrelin, which might have been inhibited by amino acids or, more plausibly, by GLP-1 [12,46]. Measurement of the circulating levels of a wider panel of anorexigenic/orexigenic peptides in future studies might be useful to solve this issue. This search (in blood samples) might produce no result considering the neuropharmacological effects of GLP-1 even within the central nervous system (CNS), with the involvement of the neural pathway of hedonic hunger, including ventral tegmental area and nucleus accumbens [43,47,48,49]. Interestingly, intake of proteins or amino acids has been demonstrated to inhibit opioid and GABAergic neurons in the nucleus accumbens, which plays a pivotal role in the rewarding connotation of feeding [12].

In the present study, the anorexigenic effects of amino acids were modest, though our results, i.e., increase in GLP-1 levels and inhibition of appetite, were statistically significant. Different reasons could be invoked to explain these results. First of all, evaluation of appetite (hunger and satiety) using the VAS method is intrinsically characterized by wide intra- and intersubject variability, so some biological differences may not be statically significant [50]. Then, the doses of our amino acids (3–6 g), which were selected on the basis of nutritional notions and pharmacological safety, might be too low.

Along this view, the ineffectiveness of amino acids in reducing appetite or intake of calories in the dinner test, in which our subjects were offered an ad libitum meal, is not surprising. In fact, the administration of low doses of amino acids (second dose at T555) might be a valid reason for these negative results. Nevertheless, we cannot rule out the occurrence of a GLP-1-mediated satiating effect at a later time (i.e., at T120, as shown in the lunch test). Moreover, in the present study, during the dinner test, subjects were administered with an unpleasant (i.e., not palatable) meal, an experimental circumstance that might have triggered a neuropharmacological response abating the GLP-1-mediated satiating effect [51]. Further studies, including more appropriate experimental paradigms, are needed to demonstrate the anorexigenic properties of our amino acid mix in the context of ad libitum eating, which is the habitual condition of an obese subject living in a Western country, where “obesiogenic” food is not missing [41].

The changes in glucometabolic homeostasis found in the present study are congruent with the higher glycemic index of maltodextrins compared to amino acids [52,53]. Notably, glucagon, a well-known hyperglycemic pancreatic hormone structurally related to incretins, including GLP-1 [54], seems to be unaffected by the amino acids selected in the present study.

Before closing, some considerations should be reported. First of all, the anorexigenic effect of our amino acid mix might depend on other peripheral and/or central mechanisms different from activation of L cells and release of GLP-1 in circulation [12]. For instance, L-leucine has been reported to inhibit food intake through neural mechanisms involving mammalian target of rapamycin (mTOR) and AMP-activated protein kinase (AMPK), “energy sensors” active in the control of energy intake, at least in the arcuate nucleus of the hypothalamus [55,56], data that require further confirmation (because of a detraction of the publication by the same authors). Tryptophan is a precursor of serotonin, a neurotransmitter known to centrally inhibit energy intake [57]. These mechanisms, though intriguing, are difficult to demonstrate in a clinical setting.

Second, a different route of administration of amino acids (i.e., intraduodenal) may produce different effects due to the bypass of gastric digestion/absorption or to the intervention of microbiota present in the large intestine [12]. Selection of the best “satiating amino acids” to be administered and pharmacokinetic issues (including dose and route of administration) are equally important to establish an effective amino acid-based supplementation for obese subjects undergoing a body weight reduction program [28].

## 5. Conclusions

L-Arginine, L-leucine, L-glutamine, and L-tryptophan, when administered to obese adolescents an hour and a half before a fixed-dose meal, are capable of (modestly) reducing appetite, an effect plausibly mediated by the parallel increase in GLP-1 levels. This anorexigenic response is not evident when obese adolescents are offered an ad libitum meal. These findings might depend on the low doses of the administered amino acids. As the results of the present work are considered preliminary, having used only one amino acid mix at a single dose, further clinical studies—even preceded by some in vitro tests—should be performed with higher doses of selected amino acids “chemosensed” by L cells in order to define new promising supplemental approaches able to counteract the worrying epidemic of pediatric obesity.
